# Advancements in the Management of Synchronous Colorectal Liver Metastases: A Comprehensive Review of Surgical, Systemic, and Local Treatment Modalities

**DOI:** 10.1007/s11912-024-01548-z

**Published:** 2024-05-22

**Authors:** Beliz Bahar Karaoğlan, Diğdem Kuru Öz, Mine Soylu Araz, Cihangir Akyol, Güngör Utkan

**Affiliations:** 1 Department of Medical Oncology, Faculty of Medicine, Ankara University, 06100 Ankara, Turkey; 2 Department of Radiology, Faculty of Medicine, Ankara University, Ankara, Turkey; 3 Department of Nuclear Medicine, Faculty of Medicine, Ankara University, Ankara, Turkey; 4 Department of General Surgery, Faculty of Medicine, Ankara University, Ankara, Turkey

**Keywords:** Colorectal cancer, Liver metastases, Synchronous liver metastases, Surgery, Locoregional therapies, Chemotherapy

## Abstract

**Purpose of Review:**

This review addresses the current landscape of colorectal cancer (CRC) with a focus on liver metastases, the third most common cancer globally. It explores recent findings in treatment strategies, emphasizing the dynamic interplay between surgery, systemic chemotherapy, and local therapies for synchronous colorectal liver metastases (CRLMs).

**Recent Findings:**

Highlighting the role of advanced imaging, the review underscores the significance of contrast-enhanced MRI in surgical planning for CRLMs. Surgical resection remains a primary choice for resectable cases, with considerations for oncologic scoring systems and tumor biology. Perioperative systemic chemotherapy plays a pivotal role, especially in conversion therapy for initially unresectable CRLMs. The review also explores various local therapies, including radiofrequency ablation, microwave ablation, stereotactic body radiotherapy, hepatic arterial infusional chemotherapy, selective internal radiation therapy, and transarterial chemoembolization for unresectable cases.

**Summary:**

A comprehensive approach, integrating surgery, systemic chemotherapy, and local therapies, is crucial for managing synchronous CRLMs. Surgical resection and perioperative chemotherapy are key players, guided by considerations of tumor biology and scoring systems. For unresectable cases, local therapies offer viable alternatives, emphasizing the need for tailored treatments. Multidisciplinary collaboration among medical oncologists, surgeons, and radiologists is essential. Ongoing research will refine treatment approaches, while emerging technologies hold promise for further advancements in managing colorectal liver metastases.

## Introduction

Colorectal cancer (CRC) is the third most common cancer and the second leading cause of cancer death worldwide [1]. In recent years, with the widespread use of cancer screening programs, the introduction of molecularly targeted biological drugs, and the use of surgical and local ablative treatments in metastatic patients, mortality has decreased and survival outcomes have improved [2]. Approximately 25% of patients with CRC are metastatic at diagnosis. Research suggest that synchronous metastatic colorectal liver disease has a poorer prognosis compared to metastatic colorectal liver disease that develops metachronously [3].

The approach to treating patients with oligometastatic CRC should revolve around the potential to completely eliminate all tumor masses. Liver resection is the best option for cure and this can be achieved through surgical R0 resection, which involves the complete removal of tumors with clear margins and no microscopic residual tumor. Although a small percent of patients may be eligible for potentially curative liver resection, the long-term survival rates following surgery for colorectal liver metastases (CRLMs) have shown remarkable improvement. Retrospective analyses and meta-analyses have highlighted that individuals with CRLMs can experience a notable 5-year overall survival rate of up to 71% following resection [4–7]. However, for a subset of individuals presenting with a limited number of small lesions, surgical resection may not be suitable due to tumor location, impaired health status or insufficient future liver remnant. In such cases, non-surgical locoregional liver-directed treatments can be an alternative to initiating systemic chemotherapy as the primary therapeutic approach. These interventions can be considered either as initial treatment options or potentially after initiating systemic therapy, addressing both the primary tumor and its metastases.

This review will focus on discussing distinctive treatment methods tailored for isolated synchronous liver metastases. It will emphasize the dynamic interaction between surgical procedures, liver-targeted therapies, and systemic treatments in CRLMs.

### 1) Imaging

Imaging plays a crucial role in assessing CRLMs for surgical planning, with high-quality contrast-enhanced computed tomography (CT) and magnetic resonance imaging (MRI) being the main modalities. Particularly, contrast-enhanced MRI, utilizing hepatospecific contrast agent (gadoxetic acid) and diffusion-weighted imaging, stands out for its superior sensitivity and specificity in detecting CRLMs, especially smaller lesions and those in patients with hepatic steatosis post-chemotherapy. A recent prospective trial across international liver surgery centers assessed the clinical impact of adding hepatospecific contrast-enhanced MRI to contrast-enhanced CT for patients scheduled for local treatment based on CT findings. The trial revealed that hepatospecific contrast-enhanced MRI led to changes in the local treatment plan for 31% of patients, including adjustments to the extent of therapy and revocation of curative-intent therapy in certain cases, emphasizing the valuable contribution of hepatospecific contrast-enhanced MRI in optimizing the management of CRLMs [8, 9••].

While whole-body positron emission tomography (PET) scans have the potential to reveal extrahepatic disease that might not be apparent on traditional imaging, it’s worth noting that chemotherapy can potentially reduce the sensitivity of PET scans for detecting CRLMs due to decreased cellular metabolic activity. Despite recommendations from National Comprehensive Cancer Network (NCCN) guidelines suggesting a staging PET scan for patients with potentially surgically curable metastatic colorectal cancer, surgical decisions following initial chemotherapy should not be solely based on PET scan results within the liver [10, 11].

PET/MR has now emerged as a novel imaging tool combining metabolic imaging and the best possible anatomical imaging. Although it is not widely available due to high cost, PET/MR seems to be very favorable in detecting CRLM. Its superiority over PET/CT is due to several technical factors like digital detectors on PET/MR devices providing a more sensitive and specific imaging, longer acquisition times of simultaneous PET/MR technique and thus higher signal to background ratios and much better anatomical correlation with respiratory gated MR imaging. Despite limited data available, according to the results of the comparative studies, it can be concluded that PET/MR outperforms PET/CT in CRLM in both patient and lesion based analyses, which leads to a change in therapeutic management in a significant majority of patients [12••].

### 2) Hepatic Resection

When it comes to addressing CRLM, the treatment plan should prioritize achieving complete resection whenever possible. Current consensus suggests that CLM should be considered “resectable” if a complete R0 resection is achievable while maintaining at least a 30% future liver remnant (RLV) or a RLV to body weight ratio ≥ 0.5% [13].

Various oncologic scoring systems have been developed to guide the selection of patients suitable for hepatic resection and predict prognosis. Among these, the criteria established by Fong et al. have gained the most acceptance and are widely used in studies. According to the FONG scoring system, assigning 1 point for each of the following criteria is considered: disease-free interval from primary to metastases < 12 months, number of hepatic metastases > 1, largest colorectal liver metastasis (CRLM) > 5 cm, node positivity in the primary tumor, and carcinoembryonic antigen level > 200 ng/ml. Patients with scores between 0–2 are classified as low-risk, those with 3–4 points are considered intermediate-risk, and those with 5 points are categorized as high-risk [14]. While not definitively predicting survival, this scoring system provides prognostic information. It assists in deciding whether to initiate surgery or systemic chemotherapy for individual patients.

Although the primary goal is to remain > 1 cm resection margin, an anticipated margin of less than 1 cm should not rule out the possibility of resection. But it is also crucial to avoid positive surgical margins, as they are associated with a higher risk of local recurrence and worse overall survival. In cases where the remaining liver is deemed too small based on cross-sectional imaging volumetrics, preoperative portal vein embolization of the affected liver can be performed to increase the volume of the future liver remnant [15].

CRLMs can be removed through either anatomic resection or non-anatomic, parenchymal-sparing resection (PSR), both of which demonstrate comparable oncological survival outcomes [16]. Anatomic resections are based on the segmental anatomy of the liver, while the size of a PSR is determined by the location and size of the CRLMs. PSR preserves a larger hepatic reserve, particularly when there is a concern about chemotherapy-induced liver injury, and may increase the likelihood of re-resection in the case of hepatic recurrence.

Tumor biology stands out as one of the most crucial factors in predicting the likelihood of recurrence and long-term survival. Prognostic tools have been developed, utilizing clinicopathological characteristics to assess the risk of recurrence following resection [17–20]. Although none of these scoring systems are able to predict disease free surival, high-risk patients can be considered for an initial course of chemotherapy as a strategy to assess the tumor’s biological behavior, thus identifying the patients who might experience rapid tumor progression and preventing them from undergoing unnecessary surgical interventions.

RAS mutations signify a more aggressive tumor nature and have been linked to a higher likelihood of positive surgical margins and poorer survival outcomes following CRLMs resection. Therefore, patients with RAS-mutated CRLMs may benefit from considering anatomic resection (instead of parenchymal-sparing resection) and/or a broader surgical margin (> 1 cm) to optimize their surgical approach [21, 22]. However, Rhaiem’s data do not support this view. They found similar local recurrence rates after both anatomic and non-anatomic resections, regardless of KRAS status. The debate on resection margins for CLM continues, but it is clear that tumor biology, rather than surgical technique and margin width, guides overall decision-making and treatment selection [23]. Portohepatic lymph node metastases associated with CRLMs are no longer considered an absolute contraindication to surgery [24, 25]. Furthermore, the presence of extrahepatic disease is no longer considered an absolute contraindication to hepatic resection, provided that a complete, R0 resection of both intra- and extrahepatic disease is feasible [24, 26, 27].

In line with this, a systematic review encompassing 52 studies and 15,144 patients, including 2308 with extrahepatic disease, found that resection for CRLM in the presence of extrahepatic disease did not warrant categorical exclusion. The review revealed 3 and 5-year overall survival rates of 58% and 26% for lung, 37% and 17% for peritoneum, and 35% and 15% for lymph nodes, respectively. The combined relative risk of death by five years favored resection in the absence of extrahepatic disease, emphasizing the importance of considering R0 resection in selected patients and challenging the notion that extrahepatic disease is an absolute contraindication to resection [28].

Patients with resectable CRLMs have the options of staged or simultaneous resection and the desicion should be made to each patient’s unique circumstances. Systematic reviews and meta-analyses have shown that there is no significant difference in outcomes between these approaches [29]. Patients requiring major colon and major hepatic surgery are best served by staged operations due to the greater risk of failure to receive adjuvant chemotherapy secondary to postoperative morbidity [30•].

In the classic colorectal-first approach, the primary colorectal tumor is resected first, followed by postoperative recovery and a period of two to three months of systemic chemotherapy. Patients who present with symptoms related to the primary tumor, such as bleeding, obstruction or perforation, are recommended to undergo primary tumor resection first. For asymptomatic patients with primary CRC, the decision between simultaneous or staged resection depends on the extent of liver involvement.

Proponents of the liver-first approach in treating CRLMs argue that delaying primary colorectal tumor resection to prioritize hepatic metastasectomy, often with systemic chemotherapy, rarely impacts primary tumor resectability. In contrast, deferring hepatic metastases resection for primary colorectal tumor removal may risk advancing liver metastases to an unresectable state [31].

**Fig. 1:**
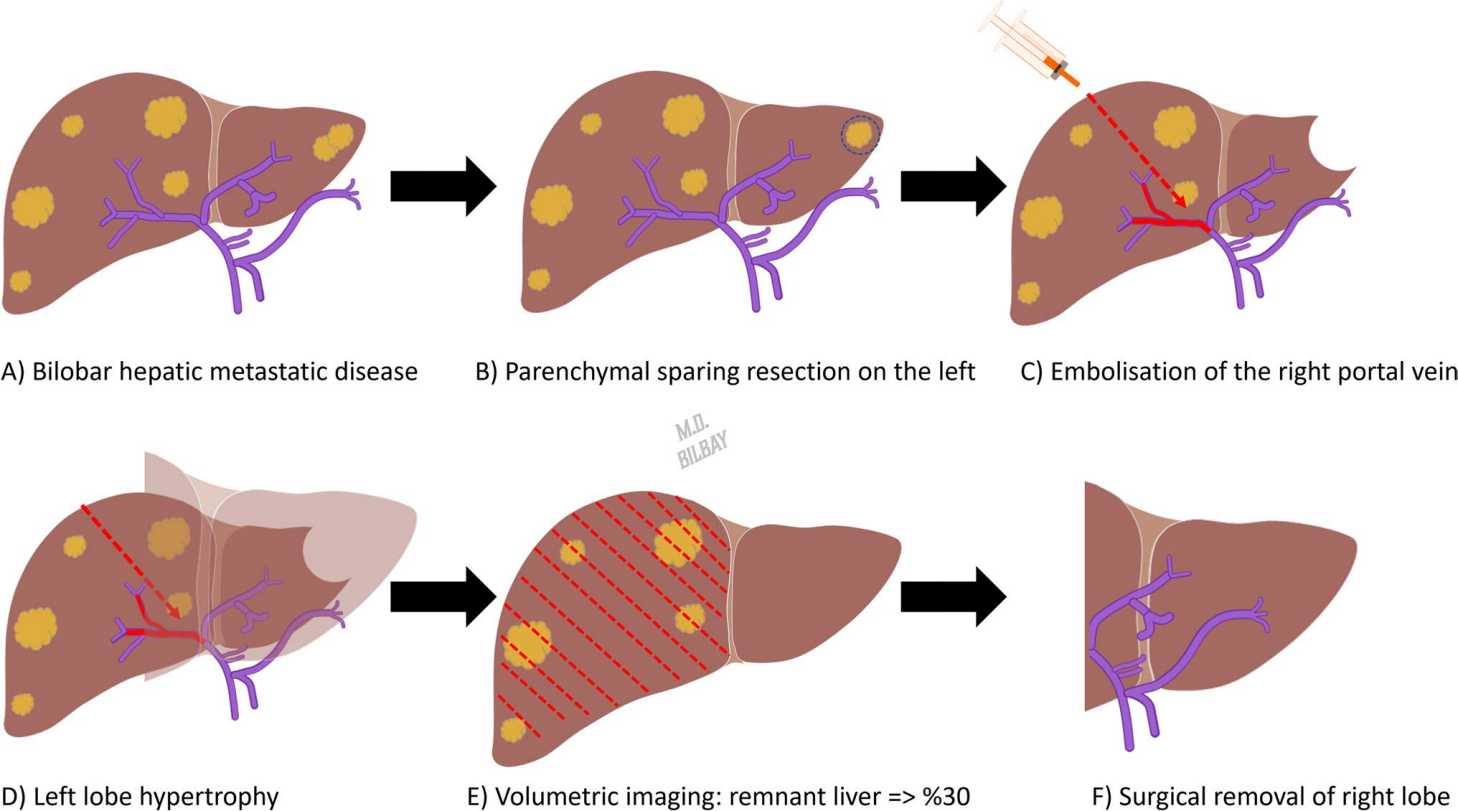
Two-stage hepatectomy procedure combined with portal vein embolization

Patients with extensive CRLMs in both liver lobes may undergo a curative resection in two stages. In the first stage, as many metastases as possible are removed during the initial colorectal primary resection. Portal vein embolization can be performed on the side with the remaining tumors to enhance liver hypertrophy while systemic therapy is administered to control the remaining disease. Once sufficient hypertrophy of the future liver remnant is achieved, the second-stage operation involves the formal anatomic resection of the remaining disease. This procedure is currently known as “associating liver partition and portal vein ligation for staged hepatectomy (ALPPS)” (Fig. 1).

### 3) Perioperatif Systemic Chemotherapy for CRLMs

**Fig. 2:**
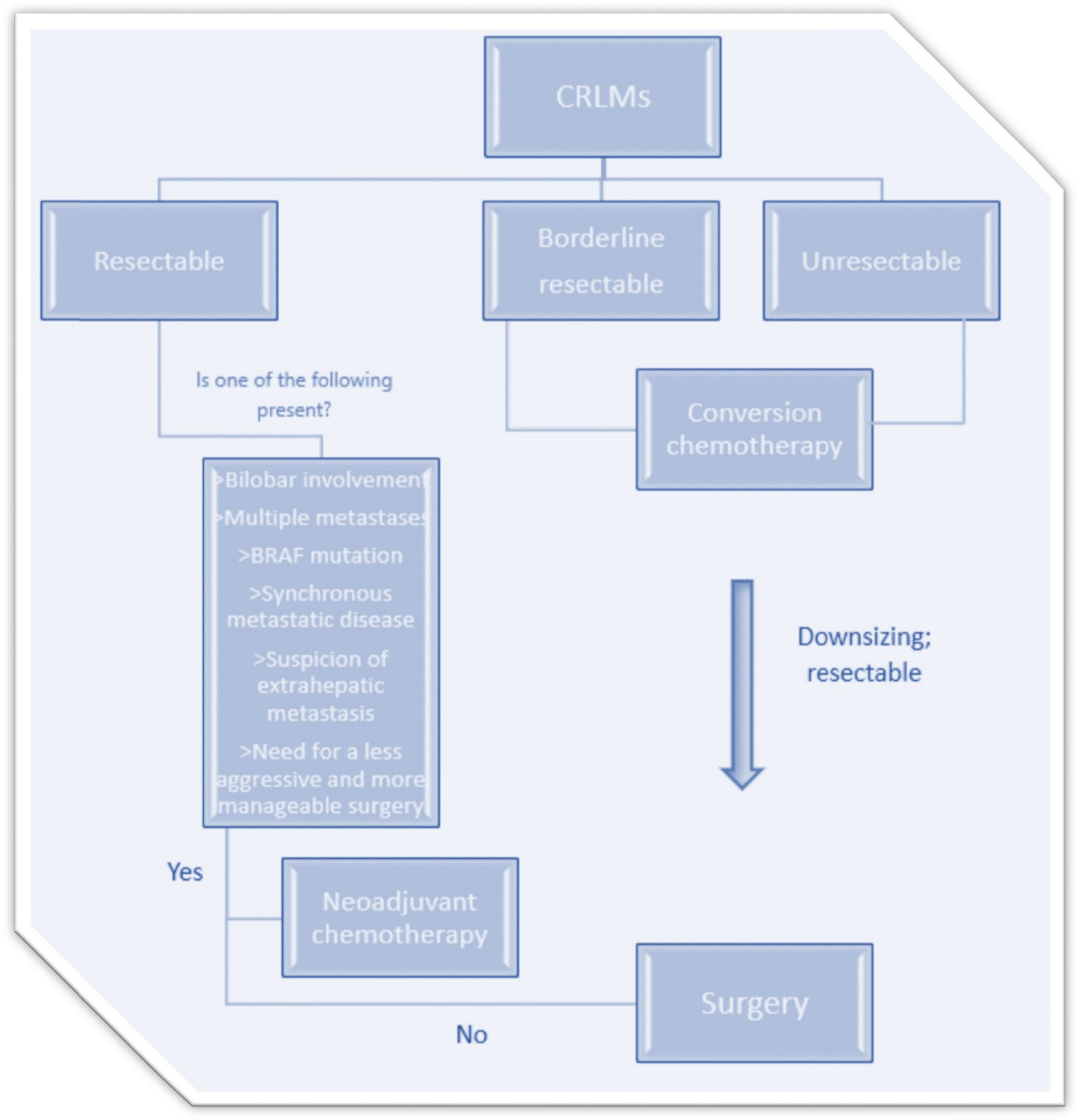
Initial Assessment of Colorectal Liver Metastases. Abbrevations: CRLM: colorectal liver metastas

The choice between perioperative chemotherapy and surgery alone, as well as the coordination of treatment sequencing, should be deliberated within a multidisciplinary team (MDT) comprising expertise in medical oncology and hepatobiliary surgery (Fig. 2).

The goal of perioperative systemic chemotherapy is to eliminate micrometastases after resection or to establish resectability in initially borderline resectable cases. In patients with a favourable risk profile and resectable CRLMs, surgery should be the primary choice, followed by adjuvant chemotherapy. For those with unfavorable risk profile, synchronous metastases, or early development of CRLM following primary tumor surgery, perioperative systemic chemotherapy should be prominently considered. When given, perioperative chemotherapy is advised for a total duration of 6 months, considering both preoperative and postoperative phases, based on findings from the EORTC 40983 trial [32].

Initial chemotherapy offers the opportunity to understand the natural progression of metastatic disease, especially crucial for patients with synchronous metastases. The concept of ’conversion therapy’ refers to the utilization of induction chemotherapy in cases of initially unresectable large or strategically positioned CRLMs into potentially resectable ones. Meta-analyses indicate a median conversion rate of 5–15%, with patients achieving resectability experiencing 5-year survival rates of 30 to 35%, significantly surpassing the expected outcomes of chemotherapy alone, where the five-year survival rate typically stands at 20% even with highly effective treatment regimens [33••].

The ideal chemotherapy regimen for conversion therapy in colorectal cancer with liver metastasis remains a subject of ongoing investigation. Doublets containing either oxaliplatin or irinotecan in combination with a fluoropyrimidine (FOLFOX or FOLFIRI) are commonly employed, with the choice influenced by the toxicity profile. Recent findings suggest that the efficacy of anti-EGFR agents varies based on the primary tumor site, with right colon cancers not benefiting from these agents for initial therapy [34–38]. Notably, the New EPOC trial, a phase 3 study, revealed a significant reduction in progression-free survival in patients with resectable colorectal liver metastasis who received cetuximab plus chemotherapy compared to those receiving chemotherapy alone. The analysis, conducted five years after recruitment cessation, demonstrated a median overall survival of 81.0 months in the chemotherapy alone group versus 55.4 months in the chemotherapy plus cetuximab group [39]. This study challenges the use of cetuximab in the perioperative setting for operable disease, emphasizing its significant disadvantage in terms of overall survival and suggesting caution in its application in this context. However, especially in borderline resectable CRLMs, the addition of cetuximab to doublet chemotherapy is recommended due to its known ability to increase resectability rates in these patients.

FOLFOXIRI is a viable option, particularly for young, healthy patients with initially unresectable liver metastases, as it offers higher rates of successful resection. In an open-label phase III trial (CAIRO5) involving nearly 300 patients with unresectable mCRC limited to the liver and a right-sided RAS/BRAF mutant primary tumor, initial therapy with FOLFOXIRI plus bevacizumab for up to six months improved progression-free survival compared to FOLFOX/FOLFIRI plus bevacizumab (11 vs. 9 months). Furthermore, the rates of complete local treatment for hepatic metastases, involving either surgery or radiation, were higher (51 vs. 37 percent) [40••].

According to the National Comprehensive Cancer Network (NCCN) guidelines, patients with synchronous initially unresectable liver metastases have several appropriate chemotherapy regimens including FOLFOX, XELOX, FOLFIRI with or without bevacizumab, infusional fluorouracil plus leucovorine (FU/LV), capecitabine with or without bevacizumab, as well as FOLFOX or FOLFIRI with or without panitumumab or cetuximab (for wild-type RAS/BRAF, left-sided tumors only), and FOLFOXIRI with or without bevacizumab. For individuals with MSI-H disease and no contraindications to immunotherapy, the consideration of immune checkpoint inhibitors (anti–PD-1, anti–CTLA-4) is warranted. This recommendation stems largely from the frontline utilization of immune checkpoint inhibitors for unresectable CRC, with KEYNOTE-177 and CheckMate 142 serving as the two primary studies guiding clinical decision-making [41–43].

An important consideration is that chemotherapeutic regimens containing irinotecan or oxaliplatin may lead to liver steatohepatitis and sinusoidal liver injury, respectively. Research has shown that chemotherapy-related liver damage, including severe sinusoidal dilatation and steatohepatitis, is linked to increased morbidity and complications in patients undergoing hepatectomy for CRLMs [44–46]. To minimize the risk of hepatotoxicity, a prudent approach involves re-evaluating patients for potential resection after two months of preoperative chemotherapy. Subsequent assessments should be conducted at intervals of 6–8 weeks, and surgery should be expeditiously performed. This strategy ensures careful monitoring and allows for timely intervention, typically approximately four weeks after the last chemotherapy session.

### 4) Local Therapies for Unresectable CRLMs

**Fig. 3:**
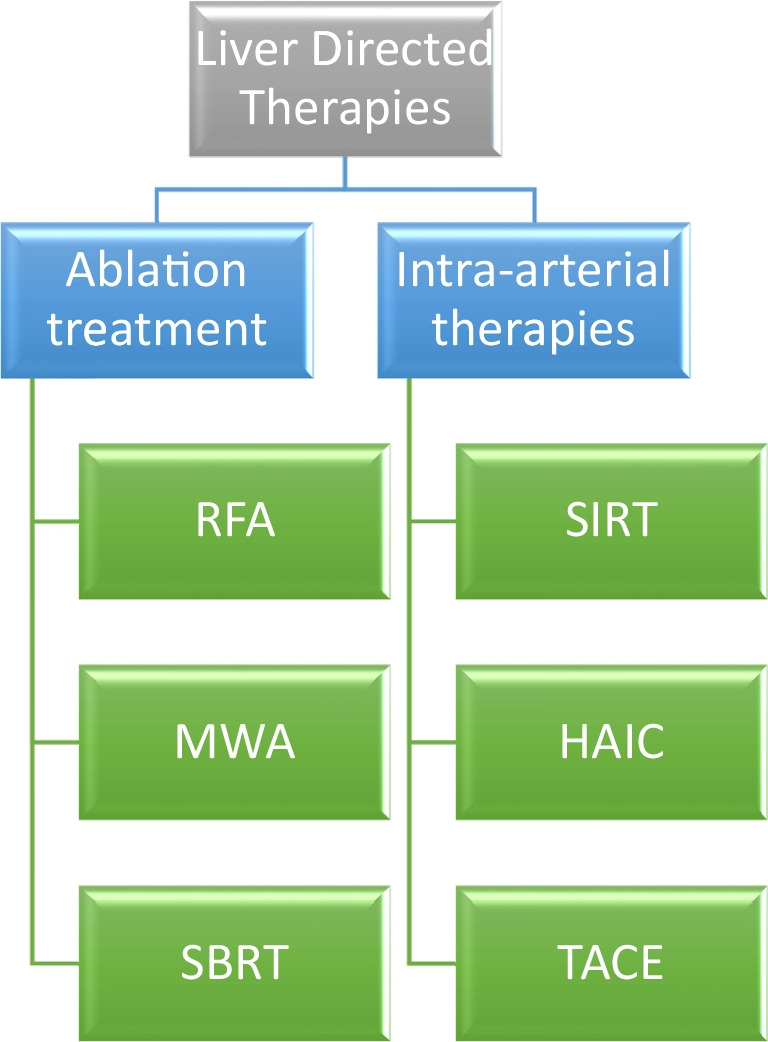
Local Treatment Options for Colorectal Liver Metastases. Abbrevations: HAIC, hepatic arterial infusion chemotherapy; MWA, microwave ablation; RFA, radiofrequency ablation; SBRT, stereotactic body radiotherapy; SIRT, selective internal radiotherapy; TACE, transarterial chemoembolisation

The chance of cure for colorectal cancer liver metastases (CRLMs) is highest with the option of surgical metastasectomy; however, only 20% of patients are considered resectable at the time of diagnosis. For patients who are not suitable for surgical treatment due to tumor location, multifocality, insufficient remnant tissue or ineligibility for surgery due to the patient’s comorbidities, or the patient’s refusal of surgery, there are local treatment options available for liver-directed therapy (Fig. 3).

Local treatments are categorized based on their technique and therapeutic goal: ablative and intra-arterial treatments. Local ablative treatments serve as curative surgical alternatives for patients ineligible for surgery, while intra-arterial treatments are often used in conjunction with systemic chemotherapy to provide local control for non-resectable and non-ablatable diseases.

Ablative options, including radiofrequency ablation (RFA) and microwave ablation (MWA), are methods categorized under thermal ablation. Within this category, there are additional choices such as cryoablation, which cools the tumor with argon infusion and inducing tissue necrosis; laser interstitial thermal therapy (LITT), causing coagulation necrosis through electromagnetic heating with laser fibers inserted into the tumor; and high-intensity focused ultrasound (HIFU), utilizing high-density ultrasound waves. However, due to the limited widespread use and insufficient level of evidence in the treatment of CRLM, this review will exclusively feature RFA and MWA. Stereotactic Body Radiation (SBRT) is a non-thermal local treatment option.

Intra-arterial treatments are designed based on the dual blood supply of the liver. In a healthy liver, 75% of the blood comes from the portal vein, and 25% comes from the hepatic artery. It is known that hepatic tumors recieve > 80% of their blood supply from the hepatic artery. Treatments delivered through the hepatic artery target tumor while relatively preserving normal parenchyma.

Local therapies assume a critical role in impeding dissemination, acting as a primary or metastasis-specific intervention, potentially obviating the need for systemic treatment, particularly in cases of slowly progressing tumors. Post-systemic therapy, local treatment transitions into a consolidative phase, strategically delaying or temporarily suspending further interventions to optimize overall therapeutic outcomes.

While surgery appears superior to local treatments in patients with resectable metastases at the time of diagnosis, the level of evidence for this comparison is limited [47, 48]. The ongoing multicenter, prospective COLLISION study is currently underway, aiming to compare ablation and surgery in resectable liver metastases of ≤ 3 cm. The results are expected to be available by 2024 [49•].

## Radiofrequency Ablation

**Fig. 4:**
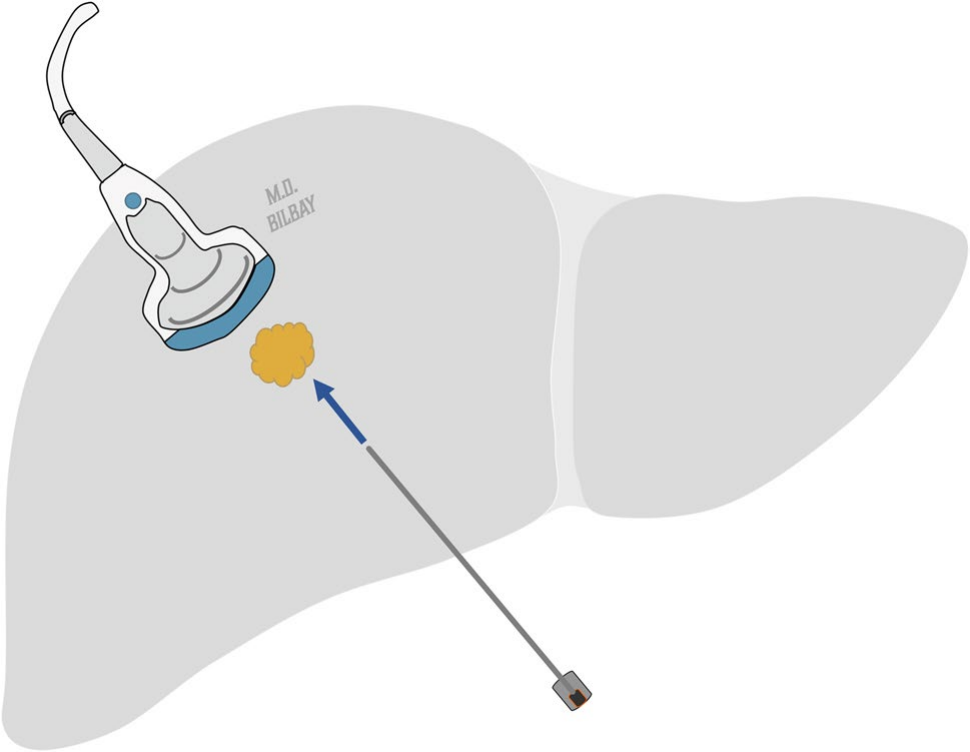
Radiofrequency ablation of CRLM

Radiofrequency ablation (RFA) is a type of thermal ablation method and aims to induce coagulative necrosis in the targeted tumor and a rim of normal hepatic parenchyma (Fig. 4). Optimal results occur when the tumor is smaller than the coagulative necrosis produced by a single ablation probe (currently around 4 cm). Success rates are highest for patients with solitary metastases or a few metastases, each smaller than 3 cm [50–52]. Lesions near large blood vessels may be inadequately treated due to the heat sink effect [53, 54]. Percutaneous RFA might be avoided for lesions near the dome or inferior liver edge to prevent diaphragmatic injury or intestinal perforation [55]. Hydrodissection is a method in which saline is instilled between the targeted tumor and neighboring structures, such as the diaphragm, to safeguard these adjacent structures from thermal injury during tumor ablation.

RF ablation can also be used in combination with metastasectomy, it is an option to perform RFA for other metastases while resectable colorectal liver metastases (CRLM) are surgically resected.

Ablation of solitary metastases yields high rates of local tumor control and survival [56]. However, literature on RFA for colorectal cancer liver metastases shows a wide range of five-year survival (14 to 55%) and local recurrence rates (3.6 to 60%). The evidence is limited and consists of a mix of patients with potentially resectable liver-isolated disease and unresectable liver metastases, with or without extrahepatic involvement [57–59].

While RFA is generally well-tolerated, it is crucial to note the potential for severe and even life-threatening complications. A common occurrence (30–40% of patients) post-RFA is the postablation syndrome, marked by symptoms such as fever, chills, pain, nausea and vomiting. This syndrome typically emerges three days after the ablation procedure, with a self-limiting nature that results in resolution within ten days [60].

Percutaneous ablation therapy efficacy is assessed via contrast-enhanced CT or MRI starting one month post-treatment. Treated tumors often display low density on CT scans, interpreted as necrosis, potentially surpassing the original tumor size.

## Microwave Ablation

Microwave ablation (MWA) is a thermal ablation method where the aim is to induce coagulation necrosis by heating the tissue through electromagnetic waves. It has gained increased usage in recent years due to its shorter ablation duration, less procedural pain, capability to treat larger tumors, and less susceptibility to the heat sink phenomenon [61]. Its side effects are similar to RF ablation but of milder intensity. While there is no randomized clinical study directly comparing radiofrequency ablation (RFA) to microwave ablation (MWA), RFA is preferred for peribiliary lesions, while MWA is favored for lesions close to large blood vessels [62, 63].

## Stereotactic Body Radiotherapy

Stereotactic body radiotherapy (SBRT) is a precise radiation technique targeting tumors by delivering a concentrated dose of radiation to targeted lesions while minimizing exposure to surrounding tissue. Particularly in elderly patients who are not suitable candidates for surgery and in those with oligometastatic disease that is technically appropriate, this approach is frequently employed with low morbidity. A meta-analysis of 18 nonrandomized studies conducted between 2006 and 2017, focusing on SBRT for patients with one to five liver metastases ineligible for surgery and mostly with prior chemotherapy, revealed promising outcomes. One- and two-year overall survival rates were 67% and 57%, respectively, while local control rates stood at 67% and 59% at the respective time points [64].

The decision between SBRT and hyperthermic ablation (RFA or MWA) typically hinges on local expertise and patient preference. Subgroup analyses in studies have demonstrated that stereotactic body radiotherapy (SBRT) provides superior local control compared to radiofrequency ablation (RFA) for tumors larger than 2 cm. However, for tumors measuring 2 cm or smaller, RFA has been shown to be superior [65]. SBRT might be favored over thermal ablation, especially for lesions adjacent to large blood vessels.

## Hepatic Arterial Infusional Chemotherapy

**Fig. 5:**
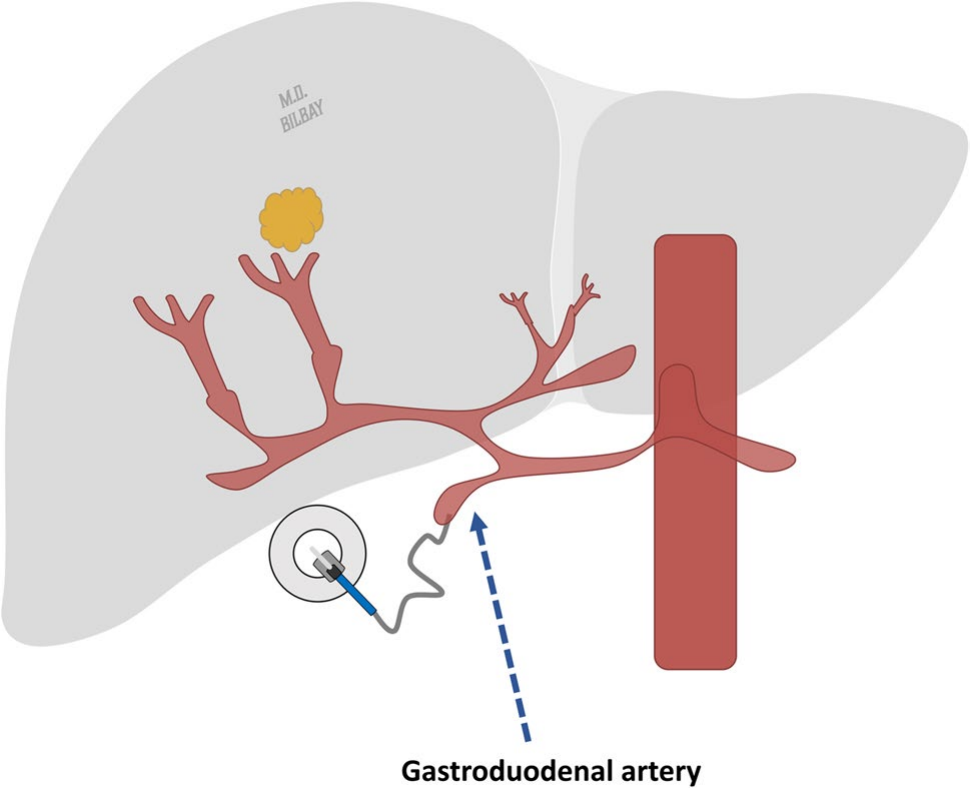
Hepatic arterial infusional chemotherapy

Hepatic arterial infusional chemotherapy (HAIC) involves surgically implanting a subcutaneous pump to administer chemotherapeutic agents directly to the liver. HAIC operates on the fundamental principle of the liver’s dual blood supply. Approximately 75% of blood flow to normal liver parenchyma is provided by the portal vein, whereas the hepatic artery contributes 25% of the blood flow. Conversely, hepatic tumors primarily derive their neovascularity from branches of the hepatic artery. HAIC pump catheters are surgically inserted into the gastroduodenal artery, enabling the delivery of arterially administered chemotherapy via the pump to attain toxic levels in tumors while comparatively preserving the normal liver parenchyma (Fig. 5) [66].

Floxuridine, a prodrug of 5-fluorouracil, stands as the most frequently utilized agent in HAI due to its advantageous pharmacokinetic properties (high rate of hepatic extraction, short half-life), effectively limiting systemic toxicity. Oxaliplatin and irinotecan are both safe and effective agents delivered via hepatic artery [67, 68].

During HAIC treatment, the idea of combining systemic chemotherapy with HAIC to achieve control over extrahepatic disease remains a subject of ongoing research. In a study investigating the impact of adding HAIC to systemic chemotherapy on disease control, the addition of HAIC to the treatment showed a survival benefit. However, the study’s non-randomized and retrospective nature diminishes the strength of the evidence [69].

Early postoperative complications are hepatic arterial injury and thrombosis, incomplete perfusion of the entire liver due to an accessory hepatic artery, misperfusion to the stomach or duodenum, or pump pocket hematoma. Late complications may involve inflammation or ulceration of the stomach or duodenum, biliary injury, pump pocket infection or catheter thrombosis.

## Selective Internal Radiation Therapy

Selective internal radiation therapy (SIRT), also known as radioembolization involves the administration of Yttrium-90, which is bound to resin or glass microspheres. These microspheres are then delivered to liver metastases through branches of the hepatic artery.

As a first line therapy, no survival benefit has been demonstrated when radioembolisation is used in conjunction with systemic chemotherapy [70••]. Although it is reported that the addition of SIRT improved OS in right-sided primary CRC compared to left-sided tumors, higher rate of adverse effects compared to chemotherapy alone led to expert guidelines recommending against the routine use of SIRT for unresectable metastatic colorectal cancer (mCRC) [71–73]. However, one should be aware of the fact that these leading studies giving directions to clinical practice guidelines included body surface area (BSA) based dosimetric approach. Further prospective studies investigating the safety and efficacy of SIRT with recent dosimetric calculations are necessary to evaluate risks and benefits of radioembolisation at first line.

Radioembolization is preferred in patients resistant to chemotherapy. There is also evidence of its benefit in PFS when used in combination with second-line systemic chemotherapy compared to patients who received chemotherapy alone [74].

Another indication of SIRT in colorectal cancer liver metastasis is its use as bridge therapy to surgery in oligometastatic metastatic disease. SIRT helps complete R0 resection and can be regarded as curative in 20–50% of patients with liver metastasis. Furthermore, SIRT can be an option in cases with potentially resectable disease but with insufficient future liver remnant [75].

Potential clinical and metabolic biomarkers of prolonged PFS or increased benefit of radioembolisation have been studied. Presence of extrahepatic disease, number of extrahepatic disease locations, serum CEA, albumin, ALT levels and tumor differentiation levels were predictors of OS. Tumor burden > 20%, Karnofsky index < 80%, CEA > 130 ng/ml or CA19.9 > 200 U/ml were also associated with OS and resection of the primary tumor was related with increased OS rates [76, 77]. Among the imaging biomarkers, pretreatment SUVmax levels was also identified as the only predictor of hepatic PFS [76]. Existence of KRAS mutation, BRAF V600E mutations, elevated microsattellite instability have also been studied as possible genetic markers of survival. However, none of these genetic parameters have been proven to be an independent predictor of survival, as many of these studies had conflicting results [78•, 79, 80].

## Transarterial Chemoembolization

Transarterial chemoembolization (TACE) is a treatment option that can be utilized in subsequent lines of therapy for chemotherapy-resistant patients. Retrospective studies have reported response rates ranging from 40 to 60% in patients with liver-dominant metastatic chemoresistant colorectal cancer [81–83].

A phase III study comparing TACE with systemic chemotherapy in patients with chemoresistant CRC with isolated hepatic metastases, demonstrated a survival benefit with TACE [84]. TACE is preferred, especially in patients with large lesions and those who are not suitable for surgery.

## Conclusion

In addressing synchronous colorectal liver metastases (CRLMs), a comprehensive approach integrating surgical, systemic, and local treatments is essential. Surgical resection stands out as a primary choice for resectable CRLMs, with the potential benefit of perioperative systemic chemotherapy to enhance overall outcomes. The pivotal role of perioperative chemotherapy, particularly in patients with unfavorable risk profiles or synchronous metastases, cannot be overstated in achieving effective disease control. For unresectable CRLMs, varied treatment options such as radiofrequency ablation (RFA), microwave ablation (MWA), stereotactic body radiotherapy (SBRT), hepatic arterial infusional chemotherapy (HAIC), selective internal radiation therapy (SIRT), and transarterial chemoembolization (TACE) offer viable alternatives. The decision-making process necessitates a thorough assessment of tumor biology, patient-specific factors, and optimized therapeutic sequencing for improved overall outcomes. While ongoing research remains crucial for refining treatment algorithms and identifying the most effective strategies in managing synchronous CRLMs in the future, the current evidence underscores the significance of a multidisciplinary approach. Involving medical oncologists, hepatobiliary surgeons, and interventional radiologists is crucial for tailoring treatments, optimizing outcomes, and improving the overall prognosis for individuals with colorectal liver metastases. As the field advances, the development of refined treatment strategies and the integration of emerging technologies are poised to further enhance the therapeutic landscape for CRLMs patients.

## Data Availability

All references utilized in the composition of this review are readily available in the literature.
